# Assessing health technology implementation during academic research and early-stage development: support tools for awareness and guidance: a review

**DOI:** 10.3389/fdgth.2024.1386998

**Published:** 2024-10-14

**Authors:** Meyke Roosink, Lisette van Gemert-Pijnen, Ruud Verdaasdonk, Saskia M. Kelders

**Affiliations:** ^1^ Department of Psychology, Health and Technology, Centre for eHealth and Wellbeing Research, TechMed Centre, Faculty of Behavioural, Management and Social Sciences, University of Twente, Enschede, Netherlands; ^2^ Department of Health Technology Implementation, TechMed Centre, Faculty of Science & Technology, University of Twente, Enschede, Netherlands

**Keywords:** health technology, innovation, implementation, support tool, medical device

## Abstract

For successful health technology innovation and implementation it is key to, in an early phase, understand the problem and whether a proposed innovation is the best way to solve the problem. This review performed an initial exploration of published tools that support innovators in academic research and early stage development with awareness and guidance along the end-to-end process of development, evaluation and implementation of health technology innovations. Tools were identified from scientific literature as well as in grey literature by non-systematic searches in public research databases and search engines, and based on expert referral. A total number of 14 tools were included. Tools were classified as either readiness level tool (*n* = 6), questionnaire/checklist tool (*n* = 5) or guidance tool (*n* = 3). A qualitative analysis of the tools identified 5 key domains, 5 innovation phases and 3 implementation principles. All tools were mapped for (partially) addressing the identified domains, phases, and principles. The present review provides awareness of available tools and of important aspects of health technology innovation and implementation (vs. non-technological or non-health related technological innovations). Considerations for tool selection include for example the purpose of use (awareness or guidance) and the type of health technology innovation. Considerations for novel tool development include the specific challenges in academic and early stage development settings, the translation of implementation to early innovation phases, and the importance of multi-disciplinary strategic decision-making. A remaining attention point for future studies is the validation and effectiveness of (self-assessment) tools, especially in the context of support preferences and available support alternatives.

## 1 Introduction

Health technology innovations can play an important role in addressing our societies' health(care) problems. However, this does require that innovations actually get developed, commercialized and implemented in (clinical) practice. In academic and early-stage development settings, it can be a challenge to oversee the complete end-to-end innovation process,^1^ to understand what is needed to get an innovation implemented in (clinical) practice, and to decide on the activities needed in projects to increase the chances of successful innovation with added value for society.

### 1.1 Implementation science perspective

Health technology innovations are not just (medical) devices^2^ or technologies “ready for deployment” (1, 2). During development, evaluation and implementation, health technologies can become innovations in *(*clinical*)* practice. For example, by creating an infrastructure for the way of working, for new services and concepts on how to change and improve healthcare, how to align work practices with technologies, how to prepare healthcare workers to use technologies, how to engage stakeholders to invest in maintenance, and how to assess the impact on healthcare (3). Within this context, implementation can be described as a process of several planned and guided activities to ultimately launch, introduce and maintain technologies in a certain context to innovate or improve healthcare (3, 4). These activities may, in addition, deliver the evidence for adoption and upscaling a technology in healthcare practices. Importantly, implementation of a certain health technology innovation may require a re-evaluation or even de-implementation of other existing health technology innovations or practices (5). Indeed, the implementation of health technology innovations is widely acknowledged as a highly complex process involving a variety of factors on multiple levels, including different stakeholders and perspectives that play a role during different innovation phases over an often-lengthy timeframe (i.e., years) (6–8). Numerous models and frameworks have evolved that aim to understand the processes and driving factors involved in innovation and implementation, and to predict outcomes, often academically framed as “implementation science” or “early health technology assessment” (3, 6, 9–11). All emphasize that implementation should be iteratively intertwined with development, involving multi-disciplinary stakeholders at an early stage. And that clear ***governance***, i.e., leadership, vision, policy and accountability, including project/innovation management, should be in place (3). However, in practice these frameworks often remain underused (9, 11, 12).

### 1.2 Failed innovation perspective

And indeed, many health technologies fail, with failure rates being reported as high as 95% (NLC—Health Ventures) (13, 14). Analyses of failed innovations have identified several critical phases in the end-to-end innovation process, the so-called “valleys of death” (13, 15, 16). A first “valley of death” occurs during the early stages of the innovation process at the transition between original scientific research and the commercialization of associated technologies (15). An important hampering factor specifically in academic settings is that the development of technologies is often primarily funded and organized for the purpose of advancing research and academic impact (15). This has important implications. For example, the innovation process often stops after the grant money has been spent, the papers have been published and the PhD student has graduated. For medical devices in particular, the complex and evolving legal and regulatory landscape both prior to as well as during commercialization, may involve more extensive clinical validation activities as well as complex, costly and lengthy market access and maintenance procedures (14, 16). This requires additional funding as compared with non-medical (health) technology innovations, but is often not covered as part of regular research funding schemes. Together, this leads to many potential innovations not reaching commercialization.

A second “valley of death” occurs at the transition between commercialization and the actual roll-out, i.e., scaling, spreading and sustained use of technology. Notably, a large portion of technology start-ups fail within the first 2–3 years after commercialization. Although there is rarely one reason for a single start-up's failure, one of the most frequently reported reasons is the lack of market (customer) need (13). This suggests that the actual challenges experienced by people that are impacted by the introduction of a health technology innovation, including changes in workflow and responsibilities, have been insufficiently assessed in previous innovation phases. Indeed, understanding the actual problem or need is closely related to understanding the complexity of implementation. The current state/best practice is constantly evolving and it should be avoided that the (wrong) wheel gets re-invented. In addition, health technologies that do not have added value for society should not be pursued up to commercialization and roll-out, especially given the constraints on the current healthcare system in terms of staff shortage, increased healthcare demand, and increased costs (17). In return, society should strive for financial systems that support the development of viable innovations that also have societal value.

### 1.3 Supporting academic research and early-stage development

There are many lessons to be learned from implementation science and from failed innovation: Early, iterative, multi-dimensional and multi-disciplinary assessment of innovation and implementation with clear governance is needed to create health technology innovations that can have added value for society, and that have a higher chance of surviving both valleys of death. However, in academic research and early-stage development settings, many organizations struggle with the question of how to best support their innovators to increase the chances of developing, evaluating and implementing health technology innovations that create impact beyond academia (7, 8, 18). Innovators' key support needs include ***awareness*** of the end-to-end innovation process and of implementation barriers/facilitators and practical ***guidance*** at the right moment in time, e.g., what stakeholders to involve when and how, what methods to employ (7, 19, 20).

At most academic institutes, generic research support is available to support health technology innovators in different phases of academic research and early-stage development. This includes for example project management and funding support. In addition, a “Knowledge Transfer Office” can support researchers with patent applications, legal advice, contracts and spin-off companies. However, innovation and implementation challenges related to the health/medical domain may require additional support, and beyond knowledge transfer. For example, support with setting up and maintaining clinical or industry collaborations, organizing clinical studies (including ethical approval), and quality and regulatory affairs (medical device classification and documentation requirements).

In addition, innovation communities have been working towards the integration or extension of models and frameworks for innovation and implementation, mostly used by experts, with practical tools for use by innovators themselves. These tools aim to support the planning, funding, and execution of health technology development in the context of the end-to-end innovation process and with the ultimate aim to implement health technology innovations in routine practice and to create socioeconomic impact (7, 21–23). Notably, some of these process support tools have become a requirement during the grant application and/or ethical approval process (1), part of healthcare insurer evaluations (24), and/or are relative requirements for the valorization of research work.

### 1.4 Considerations for tool development and selection

The present review is a follow up of previous work from our group. Specifically, a previous evaluation of health technology implementation frameworks, together with researchers and support staff, resulted in the identification of 5 implementation domains that could be useful for support tool development or selection, i.e., User, organization and system requirements; Legal requirements and ethical considerations; Effectiveness; Economic aspects; and Business plan (3, 25). As an initial validation step, the potential usefulness of these 5 domains as a support tool was tested in a retrospective case study (25). A 5-point Likert scale was used to rate the implementation “maturity”^3^ for each domain and the results were visualized in a spider-plot. The domains, scoring, and spider-plot were found to be useful in systematically assessing, rating, and visualizing implementation maturity and for identifying and discussing differences and similarities between different innovations and healthcare organizations. However, the scoring was performed by implementation experts based on subjective judgement and the study was of retrospective nature. As such, additional validation of the domains and the scoring methodology would be required prior to continuing development efforts towards a support tool for use by innovators or project/innovation managers.

In summary, there seem to be ample frameworks and tools available that could be useful in supporting the development, evaluation and implementation of health technology innovations, and this has also been the focus of several recent publications (9, 11). However, regarding the relevance of tools for supporting innovators in academic research and early-stage development with awareness and guidance, several aspects remain to be investigated. These include the number, overlap and congruence of relevant implementation domains and innovation phases, and the identification of considerations, e.g., gaps, strengths and limitations, for tool selection and/or novel tool development.

### 1.5 Objective

This review performed an initial exploration of different types of published ***tools*** that aim to support innovators (and project/innovation managers) in ***academic research*** and ***early-stage development*** settings with ***awareness*** and ***guidance*** along the end-to-end process of health technology innovation. Specifically, the aim was to identify and confirm key implementation domains and innovation phases by using a qualitative approach, as well as to discuss methodological considerations for each (type of) tool or method in order to inform tool selection and novel tool development. As such, this review provides novel directions for supporting innovators in pursuing health technology innovation and implementation as an integral part of academic research and early-stage development.

## 2 Methods

Since this concerned an initial exploration, a pragmatic non-exhaustive approach was employed. Potentially relevant tools were identified from scientific literature as well as in grey literature by non-systematic searches in public research databases and search engines, and based on expert referral. Search terms included combinations of keywords such as “implementation”, “innovation”, “tool”, “readiness”, “maturity”, “checklist”, “questionnaire”, “guidance”, and “roadmap”. Health technology innovation and implementation science experts from our institution were consulted to verify whether, based on their expertise, additional relevant tools should be considered. Tools were identified between March and August 2023.

### 2.1 Inclusion

Based on a preliminary evaluation 3 types of tools were identified for potential inclusion: (1) readiness levels, (2) questionnaires/checklists, and (3) guidance tools. Tools were considered in scope for the present review when fulfilling the following criteria:

• To be used during the development, assessment and/or evaluation of health technology innovations
• Includes key domains/milestones relevant for the end-to-end development, evaluation and implementation of health technology innovations
• (Also) targeting academic researchers/innovators
• Peer-reviewed journal paper or published grey literature from governmental agencies, non-academic organizations or innovation communities (free access only)
• Published between 2018 and 2023 and/or mandatory use in academic research (e.g., Technology Readiness Levels)
• Published in the English or Dutch language

### 2.2 Exclusion

The following exclusion criteria were employed:

• Tools associated with frameworks primarily focusing on standardizing implementation as a science, e.g., the Consolidated Framework for Implementation research (27)
• Frameworks focusing on the implementation/dissemination of research findings instead of innovations, e.g., RE-AIM (28)
• Grant-related impact tools, e.g., Impact Helper (29) or Impact Plan Approach (30)
• Tools that focus on estimating the chance of success of an innovation or project, e.g., funding success or funding potential
• Tools with a commercial purpose

### 2.3 Qualitative analysis

A step-wise approach was employed. First an overview of included tools was created. Subsequently, the overview and source documents were qualitatively analyzed, as described below, in order to identify key domains and innovation phases.

#### 2.3.1 Overview of included tools

The following items were summarized for each tool based on information sourced from the actual publication/reference: type, tool name + reference(s) + extensions (e.g., user guidance, toolkit), purpose, target user(s), domains, innovation phases, reported underlying theoretical frameworks, and other relevant findings such as methodological considerations, gaps, strengths and limitations. For each of the included tools we summarized (additional) explicit information on tool use, validation, effectiveness, and versatility across different innovation phases in the original source document and in other documents by screening citations (for academic sources) and/or by performing a google search “[(partial) Tool name or title of the source document]” AND (validation OR validity).

#### 2.3.2 Identification of key domains and innovation phases

The identification of key domains and innovation phases largely followed the approach for inductive thematic analysis, including dataset familiarization, coding/grouping (generating themes), and refining (31). For the purpose of this paper, all reported classifications, e.g., “themes”, “topics”, “risks”, or “barriers” were termed “domains”. As such “domains” cover the combined content of the reported themes, topics and risks. The previous report (3) and all newly identified tools (columns) and reported domains (rows) were arranged on a digital sheet (excel). All reported domains were compared for overlap and were grouped accordingly. Subsequently, this dataset was used to rephrase or extend the 5 domains from the previous report (3) into ***key domains***.

***Innovation phases*** concerned milestones, the timing and sequence of events, or stepwise approach. All newly identified tools that reported innovation phases (columns) and the actual reported innovation phases (rows) were arranged on a digital sheet, were compared for overlap and were grouped accordingly. Subsequently the number of different phases was reduced to a relevant minimum while still covering the end-to-end innovation process.

Reported domains or innovation phases that could not be clustered into an overarching domain or innovation phase were identified as “***implementation principles***” and were compared for overlap and grouped. Subsequently the number of implementation principles was reduced to a relevant minimum while still covering all principles.

#### 2.3.3 Mapping of tools

Lastly, all tools were mapped for coverage onto the newly framed ***key domains***, ***innovation phases*** and ***implementation principles***. For tools that did not report innovation phases, the mapping was done based on the described purpose of the tool.

## 3 Results

### 3.1 Overview of included tools

A total number of 14 tools were included for qualitative analysis in this review. The majority of tools was identified from the non-systematic search (*n* = 11), 3 tools were identified based on expert referral (18, 23, 32). Relevant details on each included tool are presented in Table 1. Tools were classified as either readiness level tool (*n* = 6) (7, 8, 32–35), questionnaire/checklist tool (*n* = 5) (6, 15, 18, 21, 23, 24) or guidance tool (*n* = 3) (4, 7, 22).

**Table 1:** Overview of included tools.

Type	Tool name *Extensions*	Intended purpose(s)	Target user(s)	Domain(s) *Classification*	Innovation phase(s)	Underlying framework(s)
Readiness level	Technology readiness level (1, 2, 33)	A method to determine the status of technology, assess risks, and make decisions concerning funding and transition of technology. A method for understanding the technical maturity of a technology during its acquisition phase. TRLs allow engineers to have a consistent datum of reference for understanding technology evolution, regardless of their technical background.	• Engineers • Researchers • Funding agencies	1. Technology	1. Basic principles observed (R) 2. Technology concept formulated (R) 3. Experimental proof of concept (R) 4. Technology validated in lab (DEV) 5. Technology validated in relevant environment (DEV) 6. Technology demonstrated in relevant environment (DEV) 7. System prototype demonstration in operational environment (DEP) 8. System complete and qualified (DEP) 9. Actual system proven in operational environment (DEP) Research (R), Development (DEV), Deployment (DEP)	Unknown
	4-axis EU framework (34)	To enlarge the scope of impact assessment of European public services through a revision and extension of the TRL scale. To assess the potential of new and existing digital technologies to promote innovation in European public services while ensuring cross-border and cross-domain interoperability. A public sector innovation policy tool to evaluate the performance of EU funded Research, Development and Innovation projects.	Not specified, however see intended purpose	1. Technology (TRL) 2. Societal (SRL) 3. Organizational (ORL) 4. Legal (LRL)	For each domain: Level 1 to Level 9, e.g., SRL1: identification of the generic societal need and associated readiness aspects. SRL6: Solution demonstrated in real world environments and in co-operation with relevant stakeholders to gain feedback on potential impacts: the society knows the solution or similar initiatives and awareness of their benefits increases. ORL7: Refinement of the roles, processes, functions and infrastructures required and retesting of the solution in relevant organizational environments. LRL2: Formulation of the need to enhance the legal normative, laws, rules and guidelines and solution concept; appraisal of legal and ethical compliance issues.	TRL (1, 2, 33)
	KTH Innovation Readiness Level^TM^ (35) *Toolkit* *User guide*	The KTH Innovation Readiness Level™ is a complete framework for guiding idea development and assessing idea status across key dimensions. It provides structure and support in the development of an early stage idea to an innovation on the market	• Teams developing ideas • Coaches or managers supporting idea development	1. Customer (CRL) 2. Technology (TRL) 3. Business model (BRL) 4. Intellectual Property Rights (IPRL) 5. Team (TMRL) 6. Funding (FRL)	For each domain: Level 1 to Level 9, e.g., CRL1: Hypothesis of possible needs in the market. CRL6: Benefits confirmed by first customer testing. BRL2: First hypothesis of possible business concept and identified overall market potential and competition. BRL4: first calculations indicating economically viable business model.	TRL (1, 2, 33)
Readiness level	Clinical readiness level (7)	To classify the readiness of medtech innovation and development projects.	• Medtech innovators • Researchers • Entrepeneurs	CRL1. Secure clinical competence in the project to complement the technical competence in the development process. CRL2. Verify and define gap/need and risk analysis. CRL3. Perform tests in a lab environment. CRL4. Perform user studies CRL5. Validate product in a clinical trial. CRL6. Validate the product's usability	1. Conceptualization 2. Concept validation 3. Product development 4. Product Launch	TRL (1, 2, 33) Manufacturing readiness level (36) KTH Innovation Readiness Level^TM^ (35)
	IDEAL-D + stage 0 (32)	To provide a universally applicable, transparent, and robust framework for planning preclinical studies derived from ethical principles which can be applied across all healthcare settings.	• Stakeholders involved in surgical and complex interventions research	Domains for stage 0 1. Classification of devices (Tier 1–3) 2. Classification of study types (Device, Patient, Clinician, System) 3. Risk-based approach (Low, Medium, High)	0. Idea (pre-clinical phase) 1. Development (first-in-human) 2. Exploration (prospective developmental studies) 3. Assessment (larger RCT or equivalent) 4. Long term follow-up (long term monitoring and registries)	IDEAL, IDEAL-D
	Medical device readiness level (8)	Technology readiness framework for communicating and planning the development of class iii medical devices.	• Medical device developers	1. Safety 2. Clinical effectiveness 3. Usability 4. Comfort 5. Affective response *Patient/healthcare worker/support staff*	1. Needs assessment 2. Prototype development. 3. Bench testing 4. Animal testing 5. Pilot testing 6. Feasibility testing 7. Pivotal testing 8. Market acceptance 9. Post-market surveillance	TRL (1, 2, 33) Human readiness level (37)
Questionnaire/checklist	Nonadoption, Abandonment, Scale-up, Spread, and Sustainability (NASSS) (6) -Complexity Assessment Tool (CAT) (21) *Questionnaire* *CAT-Short (questionnaire)* *CAT-Long (questionnaire)* *CAT-Interview* *CAT-Project*	• To inform the design of a new technology • To identify technological solutions that have a limited chance of achieving large-scale, sustained adoption • To plan the implementation, scale-up, or rollout of a technology program • To explain and learn from program failures	Questionnaire • Academic researchers • Infographics • Clinicians • Managers • Technology developers • Executive decision makers in health and care organizations • Patients and caregivers	1. Condition or illness 2. Technology 3. Value proposition 4. Adopter system 5. Organization 6. Wider context 7. Embedding and adaptation over time *Simple/complicated/complex*	Not specified, however see intended purpose	Diffusion of innovations (38) CeHReS roadmap (39) IDEAS (40) Van Dyk (41)
	Digital health Innovations applications assessment Form (18)	To assess the likelihood of a successful implementation and to ensure smooth scaling up process of their Digital Health Innovation.	• Innovators	1. Reimbursement and financing 2. Regulations and guidelines 3. Technical barriers 4. Proof of medical effectiveness 5. Economic proof of efficiency 6. User acceptance *Score: 0 (no), 1 (programmed), 2 (executed), 3 (documented)*	Not specified, however see intended purpose	Diffusion of innovations (38) WHO MAPS toolkit (42) technology acceptance model (43)
	Risk-assessment tool (13)	To bring to the constant attention the innovation risks associated with crossing the Valley of Death. The risk assessment tool poses a number of questions which lead innovation actors to generate information.	• Innovation actors managing a Valley of Death project	1. Technology concept 2. Technology performance 3. Clinical and commercial uncertainty 4. Potential impact 5. Post-valley of death commercialization strategy	The “valley of death” between foundational scientific research activity and the commercialization of the technology	Process theory (44)
Questionnaire/checklist	Healthcare innovation checklist (23)	• To assess the added value of innovations w.r.t. current care • To facilitate communication between stakeholders	• Healthcare providers • Patients • Researchers • Health care insurers • Policy makers • Innovators • Financers	1. Target group and end-user (*n* = 8) 2. Safety, effectiveness, quality (*n* = 5) 3. Preconditions (*n* = 5)	Not specified, however see intended purpose.	NASSS (6, 21) Outcomes for Implementation research (45) e-health waardenmodel (46)
	Applications and AI in healthcare checklist (24)	To prioritize digital health care innovations and to guide the decision to finance (the scaling of) digital health innovations by Dutch healthcare insurers.	• Health care insurers • Manufacturers	1. General information application and supplier 2. Risk assessment 3. End-user 4. Algorithms and AI description 5. Validation 6. Quality and affordability of care 7. Data and security aspects 8. General conditions 9. Market certification/clearance 10. Finances 11 Organizational impact 12. Platforms	1. Development 2. Validation 3. Implementation 4. Start-up/scale-up	EU Medical Device Regulations—Clinical Evaluation (47)
Guidance tool	CeHReS roadmap toolkit (4)	A practical guideline to help plan, coordinate, and execute the participatory development process of eHealth technologies. It also serves as an analytical instrument for decision making about the use of eHealth technologies and can be used for educational purposes.	• Developers (e.g., technicians, designers, and health care professionals) • Researchers • Policy makers • Education (students, health care providers)	Principles 1. Participatory Process 2. Continuous Evaluation Cycles 3. Development Intertwined With Implementation 4. Development Changes the Organization of Health Care 5. Advanced methods to assess impact	1. Multidisciplinary Project Management 2. Contextual Inquiry 3. Value Specification (incl user requirements, value specification) 4. Design (incl prototyping, business model) 5. Operationalization 6. Summative evaluation	CeHReS roadmap (39) Persuasive technology design, human-centered design, business modelling
Guidance tool	HealthTech innovation readiness innovation maturity level descriptors (22) *Guidance mapping relevant activities/deliverables for each innovation phase*	To help successfully navigate the journey from identifying and articulating an important unmet medical need to developing an innovative solution which becomes the standard of care by learning from and building on the experiences of others.	• HealthTech innovators • Budding entrepreneurs	1. Clinical 2. Market/Business 3. Technology 4. Regulatory *Risk/innovation maturity level descriptors*	1. Need (I) 2. Idea (I) 3. Proof of Concept (I) 4. Proof of feasibility (T) 5. Proof of Value (T) 6. Initial Clinical trials (T) 7. Validation of solution (T) 8. Approval & Launch (C) 9. Clinical Use (C) 10. Standard of Care (C) Invention (I), Translation (T), Commercialization (C)	TRL (1, 2, 33)
	Medtech innovation guide (7) *(web-based) guidance mapping relevant activities/deliverables for each innovation phase, including clinical readiness levels*	An integrated guide to support the innovation process within medtech research to encourage innovation, prevent interruptions of the innovation process as well as reducing the time to market. “A standalone guide to provide self-guidance in activities and give indications on support need from other actors, would serve as a tool to strengthen the control of the innovation process”.	• Medtech innovators • Researchers • Entrepeneurs	1. Clinical validation 2. Technological development 3. Business development 4. Team 5. Gender equality and equal opportunities 6. Sustainability 7. Communication 8. Funding 9. Intellectual property rights 10. Regulations and certification	1. Conceptualization 2. Concept validation 3. Product development 4. Product Launch Denotes importance of iterative development (e.g., referring to design thinking methodology)	HealthTech innovation readiness (22) End-to-end innovation adoption model (48)

For all tools, the intended purpose was made explicit in the source documentation and the target users were clarified for all but one tool (34), see Table 1—Intended Purpose. Tools intended to support the development, assessment and/or evaluation of health technology innovations (4, 6–8, 15, 18, 21, 22, 24, 32), of technology innovations in general (including health technology innovations) (33–35), or of healthcare innovations in general (including health technology innovations) (23). Notably, some tools were developed for specific health technology innovations, including for digital or eHealth innovations (4, 18, 24), applications and artificial intelligence (24), or for medical devices (7, 8, 24, 32). Several tools were developed for localized use because of reference to national rules and regulations (18), or because of the tool language (23, 24). Importantly, all tools can be used without restrictions in academic settings or during early-stage development. However, beyond academic settings, the use of the KTH Innovation Readiness Level^TM^ can be associated with trademark restrictions (35).

A wide variety of target users have been reported for the different tools including (teams or coaches of) innovators, engineers, researchers, entrepreneurs, medical device developers, managers, healthcare providers, patients, healthcare insurers, policy makers, funding agencies, and other financers, see Table 1—Target user(s).

All tools included the description of one or more domains, and innovation phases were specified for most tools, except for the Nonadoption, Abandonment, Scale-up, Spread, and Sustainability (NASSS) (6, 21), the Digital Health Innovations (DHI) applications assessment form (18), and the Healthcare Innovation checklist (23), for details see Table 1—Domain(s) and Table 1—Innovations Phase(s). A detailed analysis of domains and innovation phases is presented in the next section.

Not surprisingly, several of the included tools were based on the well-known Technology Readiness Level (TRL) (7, 8, 22, 34, 35). In addition, also other included tools, such as the NASSS (6, 21), the HealthTech Innovation Readiness (HIR) Innovation Maturity Level Descriptors (22), and the KTH Innovation Readiness Level^TM^ (35), were reported to underly the development of other included tools. Other reported frameworks that were used as a basis for tool development included various types of *theoretical frameworks* such as the Diffusion of Innovations Theory (38), the Technology Acceptance Model (43), Process Theory (44), Outcomes for Implementation Research (45), the End-to-end Innovation Adoption Model (48) and framework literature reviews (41); *readiness levels* such as the Manufacturing Readiness Level (MRL) (36) and the Human Readiness Level (HRL) (37); various types of *guidance tools* such as the CeHReS roadmap (39), mHealth Assessment and Planning for Scale (MAPS) Toolkit (42), the Integrate, Design, Assess, and Share (IDEAS) framework (40), and the e-health waardenmodel (46); *regulatory frameworks* such as the EU Medical Device Regulations—Clinical Evaluation (47); *design methodology* such as persuasive technology design, human-centered design; and *business modelling*, see Table 1—Underlying frameworks.

From the original source documents, some form of validation during tool development was described for 7 of the included tools, mostly describing expert interviews or consultations and/or case studies (4, 6–8, 18, 21, 23, 34, 39, 49). Most of the academic tools were referenced in the literature (range between 1 and 638 citations) or on google, and some extensively e.g., TRL (1, 2), NASSS (6, 21, 49), CeHReS roadmap (4, 39). For non-academic sources, no additional information was found regarding the use of the tool, except for the KTH Innovation readiness Level, which was reported to be used as the standard tool being used in their own academic population (Sweden) (35). Together, this at least suggests that some of the tools are actually used and are considered useful. However, references were generally not focused on explicitly validating the tools. Note that several tools were extended/updated after a period of use, e.g., NASSS (6, 21, 49), CeHReS roadmap (4, 39); or explicitly invited users to report on their experiences with using the tool, e.g., IDEAL-D (32), Healthcare Innovation Checklist (23) which can be considered as an additional (ongoing) validation step. Hence, ecological validation and user/expert consensus should be considered the gold standard for the evaluation of these type of tools. Effectiveness (in terms of funding or innovation success) or versatility (in terms of usefulness during different innovation phases) of the tools was not studied for any of the tools.

### 3.2 Identification of key domains, innovation phases, and implementation principles

The qualitative analysis of the included tools with respect to the reported domains and innovation phases presented in Table 1 resulted in the identification of 5 key domains, 5 innovation phases, and 3 implementation principles, for details on the analysis see “Methods”.

#### 3.2.1 Key domains

For each identified key domain a short summary and illustrative examples are presented, based on the details of the reported domains in Table 1 and the reported references.

##### Key domain 1: Understand the condition, user, organization, and healthcare system

This key domain reflects the importance of understanding and engaging with stakeholders^4^ in order to identify, evaluate, and ultimately take into account their needs, while developing, evaluating and implementing health technology innovations. This requires in the first place an understanding of the condition or illness that is being targeted (6, 21) in order to identify relevant stakeholders and experts. Regarding stakeholder needs, explicit reference was made to needs associated with the condition or illness (6, 21), to comfort (8), to safety and risk-based approach (7, 8, 24, 32), to usability (7, 8), to clinical validation (7), and to potential organizational or societal impact (e.g., adopter system, wider context) (4, 15, 24). Regarding the type of stakeholders, explicit reference was made to users or target groups (e.g., patients, healthcare workers, support staff) (8, 24, 32), customers (35), healthcare organizations and their infrastructures (6, 21, 24, 34), and societal stakeholders (e.g., political, economic, regulatory) (6, 21, 34) or “healthcare system”. Regarding the way of working, explicit reference was made to a participatory process (4), to user acceptance/user studies (7, 18), and to the embedding and adaptation of health technology innovations over time (6, 21, 35).

##### Key domain 2: Develop legal, safe, ethical, and environmentally sustainable health technology innovations

This key domain reflects the importance of taking into account additional technological, legal, safety, ethical and sustainability requirements in the context of developing, evaluating and implementing health technology (vs. other technology) innovations. Technical requirements were explicitly referenced by all tools. In addition, explicit reference was made to additional requirements for algorithms and AI description (24) and for platforms (24). Regarding legal, safety and ethical requirements, explicit reference was made to additional requirements for data and security aspects (24), gender equality and equal opportunities (7), risk assessment (7, 8, 23, 24, 32), the classification of devices (32), and regulations and certification (7, 21, 23, 24, 32). The importance of taking into account requirements for environmental sustainability of health technology innovations was explicitly referenced only by the MedTech Innovation Guide (7).

##### Key domain 3: Develop evidence strategy

This key domain reflects the importance of developing a strategy for collecting evidence (including pre-clinical and clinical evidence) during the development, evaluation and implementation of health technology innovations with the aim to demonstrate additional benefits and/or reduced risks with regards to the current state/best practice. Especially in the context of additional legal, safety and ethics requirements. Tools made explicit reference to the relevance and distinction between different study types (4, 32) or study environments (7), and to the relevance of assessing and demonstrating clinical effectiveness (8, 23), usability (7, 8), and impact or added value with respect to current (care) practices (4, 6, 15, 21).

##### Key domain 4: Investigate economic aspects

This key domain reflects the importance of investigating economic aspects relating to the development, evaluation and implementation of health technology innovations, in the context of effectiveness and impact (added value) with respect to current practices. This was reflected in the explicit mentioning of e.g., direct (implementation) costs (23), quality and affordability of care (24), economic efficiency (18), and as relevant input for business modelling (4, 7, 22).

##### Key domain 5: Develop business model

This key domain reflects the importance of developing a business model to support the development, evaluation and implementation of health technology innovations, in the context of all other key domains. Elements of business modelling that were explicitly referenced as separate domains included intellectual property rights (7, 35), potential impact (15), value proposition (4, 6, 21, 35), commercialization strategy (15), securing funding (7, 18, 24, 35), reimbursement (18), and communication (7).

#### 3.2.2 Innovation phases

The traditional technology readiness levels (TRLs) (1, 33) have been used as underlying framework for several of the included tools. As such, for each identified innovation phase the corresponding TRLs are provided as well as (partially) corresponding phases reported by other tools. For details on the reported innovation phases see references and Table 1—Innovations Phase(s).

##### Phase 1: Conceptualization

Conceptualization (7) corresponds with TRL1-2 (i.e., basic principles observed and technology concept formulated) (33). Other tools have identified (part of) this phase as ideation (22, 32), needs assessment (8), contextual inquiry (4), and/or value specification (including user requirements) (4).

##### Phase 2: Concept validation

Concept validation (7) corresponds with TRL3 (i.e., experimental proof of concept). Other tools have identified (part of) this phase as prototype development (8), design (incl prototyping, business model) (4), or proof of concept (22).

##### Phase 3: Development

Development (7, 24, 32) corresponds with TRL4-9 (i.e., technology validated in lab, technology validated in relevant environment, technology demonstrated in relevant environment, system prototype demonstration in operational environment, system complete and qualified, actual system proven in operational environment). Other tools have identified (part of) this phase as operationalization (4), bench testing (8), animal testing (8), pilot testing (8), feasibility testing (8, 22), exploration (prospective developmental studies), formative evaluation (4), proof of value (22), initial clinical trials (22), assessment (larger RCT or equivalent) (32), pivotal testing (8), validation (24), validation of solution (22), or valley of death (15).

##### Phase 4: Market access & launch

Market access & launch does no longer correspond with any TRL. Other tools have identified (part of) this phase as product launch (7), market acceptance (8), or approval & launch (22).

##### Phase 5: Post-market phase

Post-market phase does no longer correspond with any TRL. Other tools have identified (part of) this phase as implementation (24), clinical use (22), start-up/scale-up (24), long term follow-up (long term monitoring and registries) (32), summative evaluation (4), post-market surveillance (8), and/or standard of care (22).

#### 3.2.3 Implementation principles

For each identified implementation principle a short summary and illustrative examples are presented. For details see references and Table 1.

##### Principle 1: Multidisciplinary development teams (including project management)

This principle refers to the importance of the development team (7, 35) in relation to gender equality and equal opportunities (7), clinical competence (7), and multidisciplinary project and risk management (4).

##### Principle 2: Continuous evaluation cycles

The principle of continuous evaluation cycles (4) refers to the importance of employing an iterative development, evaluation and implementation process. Other tools also referenced this as embedding and adaptation over time (6, 21), or “proven over time” (35).

##### Principle 3: Development intertwined with implementation

The principle of development intertwined with implementation (4) refers to the importance of early and systematic assessment of risks and factors that might influence the uptake and adoption of a health technology innovation including all of the identified key domains and across all identified subsequent innovation phases.

#### 3.2.4 Mapping of tools

As a final step, all tools have been mapped for (partial) overlap with the identified key domains, innovation phases and implementation principles, see Table 2.

**Table 2:** Mapping of tools to identified key domains, innovation phases and implementation principles.

		Key domains					Innovation phase					Implementation principles		
**Type**	**Tool name**	Understand the condition, user, organization, and system	Develop legal, safe, ethical, and environmentally sustainable health technology innovations	Develop evidence strategy	Investigate economic aspects	Develop business model	Conceptualization	Concept validation	Development	Market access & launch	Post-market phase	Multidisciplinary development teams	Continuous evaluation cycles	Development intertwined with implementation
Readiness level	Technology readiness level (1, 31)		✓				✓	✓	✓					
	4-axis (29)	✓	✓				✓	✓	✓	✓	✓			
	KHT Innovation Readiness Level^TM^ (32)	✓	✓		✓	✓	✓	✓	✓	✓	✓	✓	✓	
	IDEAL-D + stage 0 (30)	✓	✓	✓			✓	✓	✓		✓			
	Medical device readiness level (7)	✓	✓	✓			✓	✓	✓	✓	✓			
Questionnaire/checklist	NASSS (-CAT) (5, 18)	✓	✓	✓	✓	✓	✓	✓			✓	✓	✓	
	Digital health innovation assessment form (16)	✓	✓	✓	✓	✓					✓			
	Risk-assessment tool (12)	✓	✓	✓					✓					
	Health innovation checklist (20)	✓	✓	✓	✓						✓			
	Artificial intelligence checklist (21)	✓	✓	✓	✓	✓			✓	✓	✓			
Guidance tool	CeHReS roadmap toolkit (3)	✓	✓	✓	✓	✓	✓	✓	✓	✓	✓	✓	✓	✓
	HealthTech innovation readiness (19)	✓	✓	✓	✓	✓	✓	✓	✓	✓	✓			
	Medtech innovation guide + clinical readiness level (6)	✓	✓	✓	✓	✓	✓	✓	✓	✓		✓		

✓Domain/phase/principle (partially) addressed; light orange cell, misses regulatory requirements; light green cell, explicitly includes environmental sustainability; CAT, complexity assessment tool; NASSS, nonadoption, abandonment, scale-up, spread, and sustainability; note: Medtech innovation guide + clinical readiness level grouped because of single reference source and overlapping results.

Most tools (partially) addressed at least 3 key domains (*n* = 11). The number of tools (partially) addressing each key domain was highest for the key domain “Develop legal, safe, ethical, and environmentally sustainable health technology innovations (all tools), followed by “Understand the condition, user, organization, and healthcare system” (*n* = 12), “Develop evidence strategy” (*n* = 10). “Investigate economic aspects” (*n* = 8) and “Develop business model” (*n* = 7) were only (partially) addressed by about 50% of the tools.

Several tools (partially) addressed all innovation phases (*n* = 5). However, the majority of tools were intended for use during specific parts of the innovation process covering either one (*n* = 3), three (*n* = 3), or four (*n* = 2) innovation phases. Only 50% of the tools (partially) addressed the phase “Market access & launch” (*n* = 7). For details see Table 2.

Implementation principles could be mapped only for the 4 tools from which they were identified, with tools (partially) addressing either one (*n* = 1), two (*n* = 2) or all three (*n* = 1) principles.

## 4 Discussion & conclusion

This review identified and evaluated published ***tools*** that can support innovators and project/innovation managers in ***academic research*** and ***early-stage development*** settings with ***awareness*** and ***guidance*** along the end-to-end process of development, evaluation and implementation of health technology innovations in order to overcome the so called “valleys of death”. This last section will interpret the results in the context of previous work and alternative approaches, and will discuss considerations for tool selection and for novel tool development.

### 4.1 Key domains and innovation phases

Whereas previous review papers have mostly focused on the evaluation of theoretical frameworks (9, 11), this review focused on the exploration of practical tools. The number, overlap and congruence of identified ***key domains*** and ***innovation phases***, are relevant practical considerations that could subsequently impact tool selection and/or development.

This review identified ***5 key domains*** and ***5 innovation phases***, whereas the number of domains and/or innovation phases reported by individual tools ranged from 1 (1) to 12 (24) domains and from 1 (15) to 10 (22) phases, although several tools did not specify any innovation phases (5, 18, 23). Naturally, for ***awareness***, the tension lies in wanting to be complete without being too complex. This could be of particular relevance in academic and early-stage development settings. For example, a previous evaluation together with various research groups and support staff showed that 12 domains were found to be too complex for a practical tool (3). And although the use of 9 innovation phases for readiness levels seems to be accepted by researchers for the purpose of grant applications, readiness levels are generally not used beyond this purpose (34). Indeed, the many smaller validation steps within the phases of development and deployment often do not follow a clear sequential path. Rather, they occur in parallel iterations and most of the time across different research or innovation projects (4, 7, 34). Moreover, a limited number of innovation phases allows for a more straightforward mapping or translation of ***key domains*** to smaller/project activities to be defined or discussed for specific ***innovation phases*** which could be beneficial both for ***awareness*** and ***guidance***, e.g., (7). Lastly, a lot of research on health technology innovations takes place in the post-market phase of existing (commercially available) technology (e.g., eHealth). Also, the post-market phase is the phase during which many start-ups fail (13). As such, it was deemed important to include the post-market phase, even though this was not included in all existing frameworks.

Congruence and language used for describing domains and phases are relevant aspects for both ***awareness*** and ***guidance***. As such, abstract domains such as “clinical” [e.g., (7, 22)] should be avoided, since this could actually consist of very different types of activities such as “understanding the user” and “develop evidence strategy” requiring different expertise/stakeholders. In addition, ***key domains*** and ***innovation phases*** should be understandable for innovators working on different types of health innovations and jargonistic language should be avoided since this has been reported as a significant barrier for the use of implementation frameworks and tools by non-experts (12) In addition, ***key domains*** make most sense if they can be easily mapped onto specific expertise/stakeholders, e.g., patients and healthcare organizations and system representatives (key domain 1), (medical device) design and engineering representatives (key domain 2), clinical epidemiology representatives (key domain 3), early health technology assessment representatives (key domain 4), and from business development experts (key domain 5). In relation to this latter point, it is important to realize that not all health technology innovations become medical devices. As such, ***key domains*** should avoid terms such as “regulations” or “standards” but should rather reflect the core values underlying regulations and standards (i.e., delivering legal, safe, ethical, and environmentally sustainable technology). Of course, complying with regulations and standards for medical device development is seen as an important innovation and implementation barrier, and regulations and certification were explicitly mentioned in some of the tools (7, 21, 23, 24, 32). This denotes the importance of supporting researchers with understanding the potential impact of (novel) regulations such as the Medical Device Regulations in Europe. For example in the context of timely fulfilling (ethical/legal) requirements and administration for conducting initial clinical studies. During academic research and early-stage development, this could be supported by having QA/RA expertise on board during projects or by providing regulatory-specific support tools for awareness and guidance (e.g., Lean Entries).

### 4.2 Implementation principles

Reported domains or innovation phases that could not be clustered into an overarching domain or innovation phase were identified as ***implementation principles***, mostly reflecting “the how” of implementation (research). Indeed, ***key domains*** and ***innovation phases*** provide awareness and guidance on what should be done when. However, in practice, overall project or innovation decisions generally impact all domains, and decisions need to be made regarding the relative priority of challenges and activities in a certain domain over those in another domain. These activities depend in part on the innovation phase but also on the specifics of the project. ***Implementation principles*** can provide awareness and guidance about how these aspects can be addressed in projects. For example, although each ***key domain*** asks for input from specific stakeholders, a multidisciplinary team (principle 1) needs to bring all different input together in order to intertwine implementation with development (principle 2) and make iterative decisions based on a continuous evaluation (principle 3) of the combined input. However, it should be noted that these principles were only operationalized for a selected number of tools (4, 6, 7, 21, 35), mostly by providing suggestions for team composition (4, 6, 7, 21, 35), but also by providing suggestions for step-wise evaluation using iterative research designs (4, 35), or by providing concrete guidance on identifying implementation barriers and strategies in an early development stage (4). An important aspect that was less operationalized by the included tools was ***governance*** beyond multidisciplinary project/innovation management, i.e., leadership, vision, policy and accountability. Although ***awareness*** of potential ***governance*** challenges is covered by an understanding of the healthcare system (key domain 1), it is not straightforward when and how research or innovation projects should do what to address these challenges. Some suggestions on how to organize ***governance*** are provided by frameworks describing (relatively late phase) practical implementation strategies, focusing on accountability and responsibility regarding implementation (e.g., involving an organization's executive board, recruit, designate and train for leadership, mandate change, changing liability laws) (50). During project in academic research and early-stage development settings, this could for example be supported by having implementation (science) expertise on board.

### 4.3 Considerations for tool selection

For this review three different types of tools were considered. Readiness level tools, which can be seen as milestones during innovation projects; questionnaire/checklist tools that can help to assess the complexity of a specific innovation project; and guidance tools that provide suggestions for next steps. All types of tools have their inherent pro's and con's. Some considerations were mentioned previously including the focus on specific health technology innovations (4, 18, 24); applications and artificial intelligence (24); medical devices (7, 8, 24, 32); localized use (18, 23, 24), and potential trademark restrictions (35). Still, for ***awareness*** all tools are considered to be relevant, as opposed to not using any tool. For ***guidance***, some tools may actually be less useful. Readiness level or questionnaire/checklist tools are generally easy to use and offer fixed “scoring criteria”, they do not offer much support for what to do next, with some exceptions, e.g., (35). In addition, being mostly evaluative tools, questionnaire/checklist tools may have limited relevance in early innovation phases, the NASSS being an exception (6, 21). Indeed, the need for **guidance** is likely to depend on the specific innovation phase the researcher is in, as well as on the researcher's background and preferences. When discussing some of the included tools with researchers, it was observed that the level of guidance was perceived “too linear” by some, e.g., “check the box” (7, 22), and “too vague” by others, e.g., thematic methodological suggestions (4). As such, for actual guidance, support from multi-disciplinary experts and stakeholders with knowledge of the different key domains is essential.

Besides the intended purpose, awareness and guidance, several other considerations need to be taken into account when selecting a support tool (26). For example, the intended use, reliability, ease of use, and availability of (peer) support. Although most included tools were developed on the basis of well-accepted underlying frameworks or tools, there was only limited information available regarding the validation and effectiveness of support tools. Validation and establishing effectiveness in terms of funding or implementation success can be a challenge since most of these tools are process tools (rather than intervention tools) supporting processes that can occur over multiple (research) projects and years with changing development teams and stakeholders. Previous studies therefore mostly reported ecological validation and user/expert consensus. An alternative approach could be to use the tools as (comparative) interventions in order to test specific research questions, e.g., regarding user-friendliness, versatility for different innovation phases, or for different types of users/teams. This is something that is currently being pilot tested with academic users during e.g., workshops and events and could be an initial step towards more formal evaluation. In addition, experiences with support tools and other best practices should be shared via the scientific literature and via education and training initiatives in academic and early-stage development networks.

Taken together, what this paper contributes to tool selection for both individual researchers as well as for innovation communities, is on the one hand the mapping of recent support tools onto key domains, innovation phases and implementation principles, and on the other hand providing generic considerations and suggestions that may facilitate the selection process. Given that most of the included tools and most of these considerations are not specific for academic and early-stage development settings, they may also be relevant beyond these settings, e.g., for start-ups or healthcare institutions.

### 4.4 Considerations for novel tool development

An abundance of available support tools were identified in the current review and still new tools are being developed. Although the use of support tools is generally thought to improve the innovation process and increase the chance of surviving the valleys of death, as mentioned, little is known about their effectiveness. As such, novel tool development is not simply about creating a “better” tool on the basis of gaps (or opportunities) identified for currently available tools.

As introduced, in academic and early-stage development settings some domains traditionally receive limited attention (requiring awareness), but also specific challenges may be encountered (requiring guidance) (7, 22). In general, it is often difficult for innovators to effectively translate potential barriers in later innovation phases to concrete actions during the research and early development stage. Although the tools included in this review provide awareness and guidance on what needs to be done when and how, still, these tools generally do not address how to identify shortcomings needing extra attention, how to decide on the most relevant implementation domains to address during a particular project and what would be the step-wise approach, i.e., strategy. As such, an overarching strategic tool that helps multi-disciplinary teams and stakeholders to understand, discuss, balance and set priorities over the course of their innovation project seems a relevant addition to existing tools. Such a “deliberation tool” that supports 360 degrees and end-to-end perspectives on innovation and implementation, could also help innovation projects to, after prioritization, select the most appropriate framework, methodology, or support tools for addressing implementation throughout the project. In addition, a “deliberation tool” could serve as an “impact tool”. That is, it could support early discussions on (discontinuation due to lack of) added value, and on the potential need for de-implementation of existing health technology innovations. This may be used to inform subsequent funding, commercialization and reimbursement decisions, thereby potentially reducing the number of non-value adding innovations being developed.

In its most basic form, a “deliberation tool” or “Health Technology Innovation 360” could be developed from the key domains, innovation phases, implementation principles, available support tools, and considerations for tool selection and tool development identified in this review. For example by mapping domains to innovation phases similar to the MedTech Innovation Guide (7) or the HIR Innovation Maturity Level Descriptors (22). Naturally, additional work would be needed to better understand how researchers and innovators would like to use a “deliberation tool” and whether they, for example, would require any training or additional support from experts (26). Especially in the context of anticipated repeated use, important aspects could be timing, access, and ease of use, which might be operationalized by creating an intuitive and engaging interactive digital tool [e.g., (7)]. Also the formulation of deliberation criteria, or a priority scoring and visualization thereof, may help to support ease of use and communication about the “deliberation results” [e.g., (23, 25)]. Lastly, regarding tool development, lessons might be learned from other types of multi-disciplinary collaboration processes and methods, such as risk-assessment and design-thinking/futuring (26, 51).

### 4.5 Strengths and limitations of this review

Some methodological considerations need to be taken into account when interpreting the results of this review. The current review employed a qualitative and non-systematic approach. On the one hand this may have resulted in a non-exhaustive search for and identification of support tools and associated domains and innovation phases. On the other hand this may have impacted the reproducibility of the present results. With regards to the non-exhaustiveness of the search, this was partially mitigated by also performing an expert consultation. It could be argued that a systematic (scoping) review could have been a more suitable approach. However, the purpose of this review was not to assess and maintain a(n exhaustive) list of available tools. Rather, this review aimed to validate and extend the previous work, to identify considerations for the selection and use of support tools and to inform novel tool development. With regards to the reproducibility of the findings, this is in part also warranted by the definition of clear in- and exclusion criteria specific to our research question (see Methods section). For example, in academic settings there has been an increased focus on the dissemination and translation of research results beyond academia, i.e., the publication of research results in non-scientific journals and/or the translation of knowledge into updated practice guidelines. In addition, researchers are more and more required to reflect on, plan for, and report on the broader socio-economic impact of their research. As this study was focused on supporting researchers with the ***process of health technology innovation*** in order to overcome the so-called “valleys of death”, such dissemination and impact tools were considered out of scope for this review.

Together, the qualitative and non-systematic approach is deemed appropriate given that this review was firmly rooted in theoretical frameworks and started from previous analyses that identified and pilot-tested relevant implementation domains for academic research and early-stage development settings (3, 25). The results from this paper could be used as a starting point for additional systematic assessment of practical tools similar to existing reviews on theoretical frameworks (9, 11), and may additionally include specific research questions regarding the validation and effectiveness of tools.

Another consideration is that the induction of key domains from domains reported by individual tools was complicated because some domains could be captured under multiple key domains. “Clinical” (7, 22) was already mentioned, but other examples are “post-valley of death commercialization strategy” (15), “gap/need and risk analysis” (7), “design” (4), “clinical and commercial uncertainty” (15), “value specification” (4), “potential impact” (15). This overlap in domains is a factor that complicated this analysis but also complicates the interpretation, selection, use, and development of (novel) support tools.

Lastly, the present review focused on practical tools that may support innovators (rather than implementation experts) in academic research and early-stage development settings with awareness and guidance of the end-to-end innovation process and implementation. This does, however, not mean that these tools are relevant only in the academic or early-stage development context or that the complexity of implementation can indeed be captured in a simple tool. However, tools may provide suggestions for the type of stakeholders to involve (21, 22) and for the identification of relevant themes or methods to explore, serving as a starting point for further discussion. In addition, support tools may inform and enhance other solutions for awareness and guidance. This includes academic and professional education, (improved) utilization of available experience and expertise (e.g., peer-coaching, implementing multidisciplinary teams), and building or attracting missing expertise (e.g., innovation/project management, clinical expertise, business development expertise). Support tools may also help to prepare or provide input for external support initiatives such as the innovation round-tables organized by Health Innovation Netherlands (20).

## 5 Conclusion

This review performed an initial exploration of different types of published ***tools*** that aim to support innovators with ***awareness*** and ***guidance*** along the end-to-end process of development, evaluation and implementation of health technology innovations. Specifically, it identified and key domains and innovation phases and discussed novel directions for tool selection and novel tool development. A remaining attention point for future studies is the validation and effectiveness of (self-assessment) tools, especially in the context of support preferences and available support alternatives. This review will be used to further develop the assessment and support of health technology innovation and implementation in academic research and early-stage development settings.

## Footnotes

^1^The step-wise (iterative) approach from basic (technological) concept towards an innovation that has added value and that gets implemented in routine (clinical) practice.

^2^Medical devices are a specific type of health technology subject to additional legal and regulatory requirements.

^3^Maturity with respect to the attitude and behavior of people (culture), the available processes/structures for implementation, and with respect to the integration of the technology (26).

^4^For this paper defined as a person, group or organization with a vested interest, or stake, in the decision-making and (outcome of) activities of a business, organization or project.
